# Mortality risk and mood stabilizers in bipolar disorder: a propensity-score-weighted population-based cohort study in 2002–2018

**DOI:** 10.1017/S2045796024000337

**Published:** 2024-05-23

**Authors:** Joe Kwun Nam Chan, Corine Sau Man Wong, Catherine Zhiqian Fang, Samson Chun Hung, Heidi Ka Ying Lo, Wing Chung Chang

**Affiliations:** 1 Department of Psychiatry, School of Clinical medicine, LKS Faculty of Medicine, The University of Hong Kong, Hong Kong; 2 School of Public Health, LKS Faculty of Medicine, The University of Hong Kong, Hong Kong; 3 State Key Laboratory of Brain and Cognitive Science, The University of Hong Kong, Hong Kong

**Keywords:** bipolar disorder, lithium, mood stabilizers, mortality, second-generation antipsychotics, population-based

## Abstract

**Aims:**

Accumulating studies have assessed mortality risk associated with mood-stabilizers, the mainstay treatment for bipolar disorder (BD). However, existing data were mostly restricted to suicide risk, focused on lithium and valproate and rarely adequately adjusted for potential confounders. This study aimed to assess comparative mortality risk with all, natural and unnatural causes between lithium, valproate and three frequently prescribed second-generation antipsychotics (SGA), with adjustment for important confounders.

**Methods:**

This population-based cohort study identified 8137 patients with first-diagnosed BD, who had exposed to lithium (*n* = 1028), valproate (*n* = 3580), olanzapine (*n* = 797), quetiapine (*n* = 1975) or risperidone (*n* = 757) between 2002 and 2018. Data were retrieved from territory-wide medical-record database of public healthcare services in Hong Kong. Propensity-score (PS)-weighting method was applied to optimize control for potential confounders including pre-existing chronic physical diseases, substance/alcohol use disorders and other psychotropic medications. PS-weighted Cox proportional-hazards regression was conducted to assess risk of all-, natural- and unnatural-cause mortality related to each mood-stabilizer, compared to lithium. Three sets of sensitivity analyses were conducted by restricting to patients with (i) length of cumulative exposure to specified mood-stabilizer ≥90 days and its medication possession ratio (MPR) ≥90%, (ii) MPR of specified mood-stabilizer ≥80% and MPR of other studied mood-stabilizers <20% and (iii) monotherapy.

**Results:**

Incidence rates of all-cause mortality per 1000 person-years were 5.9 (95% confidence interval [CI]: 4.5–7.6), 8.4 (7.4–9.5), 11.1 (8.3–14.9), 7.4 (6.0–9.2) and 12.0 (9.3–15.6) for lithium-, valproate-, olanzapine-, quetiapine- and risperidone-treated groups, respectively. BD patients treated with olanzapine (PS-weighted hazard ratio = 2.07 [95% CI: 1.33–3.22]) and risperidone (1.66 [1.08–2.55]) had significantly higher all-cause mortality rate than lithium-treated group. Olanzapine was associated with increased risk of natural-cause mortality (3.04 [1.54–6.00]) and risperidone was related to elevated risk of unnatural-cause mortality (3.33 [1.62–6.86]), relative to lithium. The association between olanzapine and increased natural-cause mortality rate was consistently affirmed in sensitivity analyses. Relationship between risperidone and elevated unnatural-cause mortality became non-significant in sensitivity analyses restricted to low MPR in other mood-stabilizers and monotherapy. Valproate- and lithium-treated groups did not show significant differences in all-, natural- or unnatural-cause mortality risk.

**Conclusion:**

Our data showed that olanzapine and risperidone were associated with higher mortality risk than lithium, and further supported the clinical guidelines recommending lithium as the first-line mood-stabilizer for BD. Future research is required to further clarify comparative mortality risk associated with individual SGA agents to facilitate risk-benefit evaluation of alternative mood-stabilizers to minimize avoidable premature mortality in BD.

## Introduction

Bipolar disorder (BD) is a severe mental disorder associated with significant functional disability, elevated suicide risk, increased physical morbidity and premature mortality (Chan *et al.*, 2022a; McIntyre *et al.*, 2020; Plans *et al.*, 2019). Given that it is potentially chronic in nature with relapse-remitting illness course, the majority of patients require maintenance treatment of mood stabilizers. Lithium is the first-line mood-stabilizing medication treatment for BD, and is found to be related to reduced risk of all-cause mortality and suicide (Cipriani *et al.*, 2013). In recent decades, lithium has shown a declining trend of use due to its narrow therapeutic window and potential physical complications, including hypothyroidism and renal impairment (McKnight *et al.*, 2012; Schoretsanitis *et al.*, 2022), and has been partially replaced by second-generation antipsychotics (SGA) and anticonvulsants (especially valproate) (Bellivier *et al.*, 2014; Lyall *et al.*, 2019; Rhee *et al.*, 2020). Research assessing mortality risk associated with these alternative mood stabilizers is warranted to inform clinical decision on treatment regimens in BD.

Accumulating research has examined the association of mood stabilizers with mortality risk in BD. An earlier meta-analysis of randomized controlled trials demonstrated superior efficacy of lithium in reducing suicide risk relative to placebo, but no significant difference compared to valproate, olanzapine and quetiapine in patients with unipolar/bipolar depression (Cipriani *et al.*, 2013). An updated meta-analysis, however, reported that lithium did not differ from placebo or treatment-as-usual in suicide rate reduction (Nabi *et al.*, 2022). Mixed results were observed in recent register-based cohort studies in this respect. Some showed that lithium, but not valproate or antipsychotics, was associated with decreased suicide risk (Song *et al.*, 2017; Toffol *et al.*, 2015), while others reported favourable results for both lithium and valproate (Antolín-Concha *et al.*, 2020; Chen *et al.*, 2023; Tsai *et al.*, 2016), compared to non-treatment. Some (Antolín-Concha *et al.*, 2020; Crump *et al.*, 2013; Song *et al.*, 2017), albeit not all (Hayes *et al.*, 2016), head-to-head medication comparison studies showed that lithium was associated with reduced suicide rate relative to valproate. Discrepant findings were also reported on all-cause and natural-cause mortality risk in relation to mood stabilizers. A Taiwanese study revealed reduced all-cause and natural-cause mortality associated with lithium and valproate, compared to no mood-stabilizer treatment (Chen *et al.*, 2023). A Finnish study demonstrated no association of elevated all-cause mortality with lithium and valproate (Toffol *et al.*, 2015), while a US study found a lower non-suicide mortality rate in lithium-treated BD patients than in valproate-treated counterparts (Smith *et al.*, 2015).

Notably, existing population-based cohort studies assessing association of mood stabilizers with mortality risk in BD were hampered by several important methodological limitations. First, most studies focused on suicide outcome only (Antolín-Concha *et al.*, 2020; Hayes *et al.*, 2016; Oquendo *et al.*, 2011; Song *et al.*, 2017; Toffol *et al.*, 2015), and there is limited research evaluating natural-cause mortality which, nonetheless, accounted for the majority of death causes (up to two-thirds) in BD (Chan *et al.*, 2021a; Paljärvi *et al.*, 2023). Second, past studies did not take into consideration the effect of concomitant or sequential use of multiple mood stabilizers and other psychotropic medications, which might confound the study results (Chen *et al.*, 2023; Lin *et al.*, 2023; Toffol *et al.*, 2015). Third, previous studies primarily restricted their analysis to lithium, valproate and broadly categorized antipsychotics (Antolín-Concha *et al.*, 2020; Smith *et al.*, 2015; Song *et al.*, 2017; Toffol *et al.*, 2015). However, literature conducted in schizophrenia patients indicated differential relationships between all-cause mortality risk and individual antipsychotics (Taipale *et al.*, 2020). Thus far, no studies have systematically examined the risk of all-cause, natural-cause and unnatural-cause mortality associated with commonly prescribed individual SGAs in BD patients.

We have previously conducted a population-based cohort study to assess premature mortality in BD patients, utilizing a territory-wide medical-record database of public health service in Hong Kong (HK), a metropolitan city located at the south-eastern tip of China, with total population of over 7.5 million. Our results demonstrated that BD patients had 2.6-fold elevated all-cause mortality rate and approximately 7 years of shorter lifespan relative to the general population (Chan *et al.*, 2021a). In the current investigation, we used the same study cohort and aimed to comprehensively evaluate the comparative mortality risk of all-cause, natural-cause and unnatural-cause deaths between mood stabilizers of lithium, valproate and three most commonly prescribed SGAs (i.e., quetiapine, risperidone and olanzapine) in patients with first-diagnosed BD over a 17-year period, taking into account important potential confounders including pre-existing chronic physical diseases, substance/alcohol use disorders and other psychotropic medications. Propensity-score (PS)-weighted models were employed to further optimize covariate adjustment.

## Methods

### Data source

Data of the patient cohort were extracted from the Clinical Data Analysis and Reporting System (CDARS; Hospital Authority Head Office IT Department, 2003), a territory-wide electronic health-record database developed by the Hospital Authority (HA) which is a statutory body delivering government-subsidized, universal health coverage to all HK residents (approximately 92% being Chinese) by managing all public hospitals, specialist and general outpatient clinics in HK. Detailed description of CDARS has been reported elsewhere (Cheung *et al.*, 2007). Briefly, CDARS is an integrated, longitudinal patient electronic record system capturing clinical data across all healthcare settings of HA facilities. The database contains patients’ demographics and clinical information including diagnoses, attendances to outpatient clinics and emergency departments, hospital admissions and prescribing/dispensing records of medications. Data on dates and causes of death were retrieved from CDARS via internal linkage to regional death registries from the Immigration Department. Patients’ death status was also directly recorded and verified by CDARS as the vast majority of deaths in HK occur in public hospitals, thereby facilitating accurate ascertainment of death. Clinical data are collected and entered into computerized clinical-management system by treating clinicians and other healthcare professionals, and are then transferred to CDARS for audit and research purposes. CDARS generates unique, anonymized patient identifiers to protect privacy and to link all medical records. This database has been used to conduct high-quality population-based studies on psychiatric disorders including schizophrenia and BD (Chan *et al.*, 2021b, 2021a, 2022b; Chang *et al.*, 2020; Yung *et al.*, 2021, 2020) and pharmacoepidemiological investigations (Chai *et al.*, 2022; Hung *et al.*, 2023; Kan *et al.*, 2022; Law *et al.*, 2023).

### Study population

We identified all individuals aged ≥15 years who received a first-recorded diagnosis of BD for public psychiatric inpatient or outpatient treatment in HK between 1 January 2002 and 31 December 2018. Diagnosis of BD was recorded and ascertained by the International Classification of Diseases, 10th revision (ICD10 codes: F30 and F31). Diagnostic ascertainment took into consideration the longitudinal illness course (Chang *et al.*, 2009) and individuals with their diagnosis changed to schizophrenia or schizoaffective disorder (ICD10 codes: F20 and F25) before the end of follow-up (as their mostly recently assigned principal diagnosis) were excluded. Patients were followed up from the date of their first-recorded BD diagnosis until the date of death or 31 December 2018, whichever came first. The study was approved by the Institutional Review Board of the University of Hong Kong/Hospital Authority Hong Kong West Cluster. Since individual patient records in this database were completely unidentifiable, no informed consent was required.

### Exposure to mood stabilizers

Five mutually exclusive mood-stabilizer exposure groups including lithium, valproate, quetiapine, risperidone and olanzapine were derived based on medication records of included BD patients. Quetiapine, risperidone and olanzapine represented the three most frequently prescribed SGAs in the study cohort (Supplementary Table S1) as the treatment for BD, and were thus selected in the subsequent comparative analyses on mortality risk. BD patients who had been prescribed with one of the five specified mood stabilizers only during study period were classified as users to that mood stabilizer. For patients who had received more than one of the studied mood stabilizers during the study period, their group membership status was determined by the prescribed mood stabilizer with the longest cumulative exposure duration within the study follow-up. BD patients who had not exposed to any of the five studied mood stabilizers were excluded from the analysis.

### Outcomes

The main outcome measure was all-cause mortality. Causes of death were also classified according to ICD10 codes, and were divided into natural and unnatural causes. Natural causes comprised infectious and parasitic diseases (A00–B99), neoplasms (C00–D48), metabolic diseases (E00–E90), neurological diseases (G00–G99), cardiovascular diseases (I00–I99), respiratory diseases (J00–J99), digestive diseases (K00–K93) and genitourinary diseases (N00–N99). Unnatural causes included accidents (V01–X59), self-harm (X60–X84) and other external causes (X85–Y98). Patients with unknown death causes were excluded in analyses for mortality of natural and unnatural causes.

### PS weighting and covariates

A PS weighting model was performed to limit confounding in the five exposure groups of mood stabilizers. Taken into consideration the availability of clinical information adequately captured in the database, an array of preselected candidate covariates comprising patients’ demographics (sex, age at first-recorded diagnosis of BD, catchment areas of healthcare service), and pre-existing chronic physical diseases (i.e., physical morbidity burden) as quantified by Charlson Comorbidity Index (Charlson *et al.*, 1987; Deyo *et al.*, 1992) as well as the presence of epilepsy, diabetes, hypertension and dyslipidaemia, comorbid substance and alcohol use disorders, history of past psychiatric admission (as a proxy for illness severity) and the use of psychotropics other than the five specified mood stabilizers during the study period including mood-stabilizing anticonvulsants (lamotrigine, carbamazepine), antipsychotics (first-generation antipsychotics and SGAs other than the three specified SGAs) and antidepressants. Details of diagnostic codes for physical and psychiatric morbidities are listed in Supplementary Table S2. Multinomial PS weighting was obtained using generalized boosted models (McCaffrey *et al.*, 2013). We sought to estimate the average treatment effect for the PS weighting among the five exposure groups, based on the premise that their membership assignment was an exchangeable option (Desai and Franklin, 2019). We took the maximum absolute standardized mean difference (ASMD) across exposure groups in each covariate as the diagnostic measure of between-group balance, where ASMD >0.20 denotes notable group differences. Before weighting, a total of 16 (out of 27) comparisons in covariates showed ASMD >0.20 and was reduced to 1 after weighting (Supplementary Figure S1). The imbalanced covariate was additionally adjusted in the PS-weighted regression model to control for remaining imbalances among exposure groups.

### Statistical analysis

Demographics, baseline physical comorbidities, substance and alcohol use disorders and prescription profile within follow-up of BD patients among five mood-stabilizer exposure groups were compared. Incidence rates for mortality due to all-, natural- and unnatural-causes among five exposure groups at follow-up were estimated by an exact 95% confidence intervals (CIs) based on a Poisson distribution. PS-weighted Cox proportional-hazards regression models were conducted to evaluate the relative risk of mortality among five exposure groups, with lithium-treated group as the reference category. Kaplan–Meier curves were used to visualize the survival rates of each exposure group. To explore the potential influence of length of exposure to studied mood stabilizers on mortality risk, the analyses were repeated in BD patients of each of the five mood-stabilizer exposure groups with cumulative exposure to the specified mood stabilizer ≥90 days, ≥180 days, ≥365 days and ≥730 days (2 years). We then specifically restricted the analyses to patients with short exposure duration of <90 days, <180 days and <365 days, to the specified mood stabilizers in each exposure group and examined the relationship of short mood-stabilizer exposure duration with mortality risk. Three sets of sensitivity analyses were conducted. First, only patients with the length of cumulative exposure to the specified mood stabilizer ≥90 days (i.e., cumulative exposure to lithium in the lithium-treated group; cumulative exposure to valproate in the valproate-treated group and so forth) and its medication possession ratio (MPR) ≥90% were included to ensure sufficient exposure to the specified mood stabilizer in each exposure group. MPR of the specified mood stabilizer was calculated by dividing the length of its cumulative exposure by the total observation time at follow-up. Prior research has shown that medication prescription data were adequately captured and reliably recorded in CDARS (i.e., electronic medical-record database used in the current study), and were used to generate medication exposure-related parameters including cumulative exposure duration and MPR (Man *et al.*, 2017). Second, MPR of the specified mood stabilizer and other studied mood stabilizers in the exposure group was calculated. We only included patients with MPR of the specified mood stabilizer ≥80% and MPR of other studied mood stabilizers <20% in the analyses to reduce confounding effect of the other studied mood stabilizers in each exposure group. Third, monotherapy analysis was conducted by including patients who had been prescribed with only one of the five specified mood stabilizers within the entire follow-up. The proportional-hazards assumptions for all analyses were confirmed using log-minus-log plot (Kleinbaum and Klein, 2012). Results of all Cox proportional-hazards regression models were presented as hazard ratios (HRs) in 95% CIs. All statistical analyses were performed using R (version 4.0.2). Generalized boosted model was implemented with the *twang* package (Ridgeway *et al.*, 2012). *P* < 0.05 was considered statistically significant.

## Results

### Characteristics of the study sample

A total of 12,797 BD patients (mean age = 41.3 years, SD = 15.4) were identified within the study period by the medical-record database. In this whole BD cohort, 3701 patients were excluded as their BD diagnosis was not incident in nature within the study period. Among the incident BD cohort (*n* = 9094), 957 patients who were not prescribed with any of the five studied mood stabilizers within the study period were excluded from the subsequent analyses, resulting in a total of 8137 patients as the final study cohort (mean age = 39.2 years, SD = 15.4). The mean duration of follow-up for BD patients was 7.2 years (SD = 4.7). A total of 488 deaths was recorded in the overall sample, of which 358 (73.4%) had known causes. There were 1027, 3580, 797, 1975 and 757 patients assigned to lithium, valproate, olanzapine, quetiapine and risperidone exposure groups, respectively. Lengths of cumulative exposure per mood stabilizer in each of the five studied mood-stabilizer exposure groups are shown in Supplementary Table S3. Patients in the lithium-treated group tended to be younger and had lower prevalence of hypertension, dyslipidaemia, diabetes and substance use disorder than those in the other exposure groups (Table 1).

**Table 1.** Characteristics of mood-stabilizer exposure groups

	Exposure groups					
Characteristics	Lithium (*n* = 1028)	Valproate (*n* = 3580)	Olanzapine (*n* = 797)	Quetiapine (*n* = 1975)	Risperidone (*n* = 757)	*p* value^a^
Mean age, years (SD)	36.4 (13.7)	39.7 (15.7)	36.3 (14.3)	40.8 (15.7)	39.3 (15.5)	<0.001
Female sex, *n* (%)	577 (56.1)	2080 (58.1)	447 (56.1)	1356 (68.7)	409 (54.0)	<0.001
Medical comorbidities^b^, *n* (%)						
Hypertension	25 (2.4)	229 (6.4)	29 (3.6)	148 (7.5)	58 (7.7)	<0.001
Diabetes mellitus	15 (1.5)	134 (3.7)	14 (1.8)	84 (4.3)	40 (5.3)	<0.001
Dyslipidaemia	11 (1.1)	116 (3.2)	15 (1.9)	95 (4.8)	36 (4.8)	<0.001
Epilepsy	5 (0.5)	33 (0.9)	9 (1.1)	19 (1.0)	7 (0.9)	0.634
Alcohol use disorder	5 (0.5)	28 (0.8)	3 (0.4)	21 (1.1)	6 (0.8)	0.297
Substance use disorder	10 (1.0)	50 (1.4)	10 (1.3)	45 (2.3)	9 (1.2)	0.028
Charlson comorbidity score (CCI)^c^, mean (SD)	0.7 (1.0)	1.0 (1.5)	0.7 (1.2)	1.1 (1.5)	1.1 (1.6)	<0.001
History of psychiatric hospitalization, *n* (%)	790 (76.8)	2610 (72.9)	593 (74.4)	1215 (61.5)	537 (70.9)	<0.001
Prescription profile^d^, *n* (%)						
Lithium	-	742 (20.7)	206 (25.8)	374 (18.9)	109 (14.4)	<0.001
Valproate	503 (48.9)	-	418 (52.4)	972 (49.2)	371 (49.0)	<0.001
Other anticonvulsants^e^	223 (21.7)	519 (14.5)	153 (19.2)	477 (24.2)	96 (12.7)	<0.001
Olanzapine	412 (40.1)	1085 (30.3)	-	343 (17.4)	90 (11.9)	<0.001
Quetiapine	555 (54.0)	1087 (50.5)	326 (40.9)	-	191 (25.2)	<0.001
Risperidone	387 (37.6)	1462 (40.8)	309 (38.8)	469 (23.7)	-	<0.001
Other SGA^f^	246 (23.9)	827 (23.1)	223 (28.0)	387 (19.6)	133 (17.6)	<0.001
FGA	548 (53.3)	1790 (50.0)	252 (31.6)	535 (27.1)	251 (33.2)	<0.001
Antidepressants	555 (54.0)	2068 (57.8)	379 (47.6)	1223 (61.9)	349 (46.1)	<0.001

*Note*: FGA, first-generation antipsychotics; SD, standard deviation; SGA, second-generation antipsychotics.

a Chi-square and one-way ANOVA tests were conducted for analysis of categorical and continuous variables, respectively.

b At least one medical comorbidity was measured at baseline.

c Charlson Comorbidity Index (CCI) score is a widely used and well-validated measure for physical morbidity burden (Charlson *et al.*, 1987; Deyo *et al.*, 1992), which assesses the presence of 17 chronic physical diseases including cerebrovascular disease, hemiplegia or paraplegia, myocardial infarction, congestive heart failure, peripheral vascular disease, respiratory diseases, peptic ulcer disease, mild liver disease, moderate or severe liver disease, renal diseases, rheumatological diseases, malignancy without metastasis, metastatic solid tumour, diabetes metabolic disturbances, diabetes with chronic complications, AIDS or HIV and dementia. In the current study, age-adjusted adapted CCI was computed. As diabetes was a disease of interest, it was evaluated separately and excluded from CCI score calculation. The higher the CCI score, the greater the physical comorbidity burden (in terms of the number and severity of physical multi-comorbidity).

d Medications could be prescribed concomitantly or sequentially during study follow-up.

e Other anticonvulsants included prescriptions of carbamazepine or lamotrigine.

f Other second-generation antipsychotics (SGA) included any prescription of clozapine, amisulpride, paliperidone, aripiprazole, lurasidone, ziprasidone, sertindole.

### All-cause, natural-cause and unnatural-cause mortality risk and mood stabilizers

As shown in Table 2, the incidence rate per 1000 person-years were 5.9 (95% CI: 4.5–7.6), 8.4 (7.4–9.5), 11.1 (8.3–14.9), 7.4 (6.0–9.2) and 12.0 (9.3–15.6) for all-cause mortality in lithium, valproate, olanzapine, quetiapine and risperidone exposure groups, respectively. PS-weighted Cox regression models indicated significantly elevated risk of all-cause mortality associated with olanzapine (HR: 2.07 [95% CI: 1.33–3.22]) and risperidone (1.66 [1.08–2.55]), relative to lithium.

**Table 2.** Mortality risk of mood-stabilizer exposure groups

	Exposure groups				
Variables	Lithium (*n* = 1028)	Valproate (*n* = 3580)	Olanzapine (*n* = 797)	Quetiapine (*n* = 1975)	Risperidone (*n* = 757)
Person-years at risk (PYAR)	9360.1	30,007.8	4044.8	10,787.0	4732.8
Mean years of follow-up (SD)	9.1 (4.9)	8.4 (4.6)	5.1 (4.1)	5.5 (4.1)	6.3 (4.3)
All-cause death					
Number of events	55	251	45	80	57
Incidence rate per 1000 PYAR (95% CI)	5.9 (4.5–7.6)	8.4 (7.4–9.5)	11.1 (8.3–14.9)	7.4 (6.0–9.2)	12.0 (9.3–15.6)
Unadjusted HR (95% CI)	Reference	1.44 (1.08–1.93)	1.96 (1.32–2.92)	1.31 (0.93–1.85)	2.13 (1.47–3.08)
Adjusted HR^a^ (95% CI)	Reference	1.10 (0.79–1.53)	2.07 (1.33–3.22)	0.91 (0.62–1.35)	1.66 (1.08–2.55)
Natural death					
Number of events	20	139	24	44	25
Incidence rate per 1000 PYAR (95% CI)	2.2 (1.4–3.4)	4.7 (4.0–5.6)	6.0 (4.1–9.0)	4.1 (3.1–5.6)	5.4 (3.6–8.0)
Unadjusted HR (95% CI)	Reference	2.25 (1.41–3.59)	3.21 (1.77–5.83)	2.18 (1.28–3.71)	2.76 (1.53–4.97)
Adjusted HR^a^ (95% CI)	Reference	1.32 (0.75–2.31)	3.04 (1.54–6.00)	1.20 (0.65–2.25)	1.50 (0.76–2.99)
Unnatural death					
Number of events	14	53	10	17	23
Incidence rate per 1000 PYAR (95% CI)	1.5 (0.9–2.6)	1.8 (1.4–2.4)	2.6 (1.4–4.8)	1.6 (1.0–2.6)	5.0 (3.3–7.5)
Unadjusted HR (95% CI)	Reference	1.17 (0.65–2.10)	1.44 (0.64–3.25)	0.92 (0.45–1.88)	2.95 (1.52–5.75)
Adjusted HR^a^ (95% CI)	Reference	1.16 (0.632.16)	1.52 (0.63–3.65)	0.93 (0.43–2.00)	3.33 (1.62–6.86)

*Note*: CI, confidence interval; HR, hazard ratio; SD, standard deviation.

a The model was propensity-score weighted with additional adjustment for concomitant/ sequential prescription of studied mood stabilizers other than the specified one.

**Figure 1.**
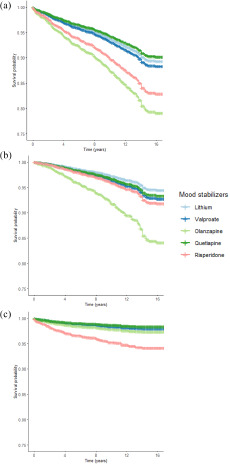
Survival curves for mood-stabilizer exposure groups: (a) all-cause deaths; (b) natural-cause deaths and (c) unnatural-cause deaths.

For natural-cause mortality, the incidence rates per 1000 person-years were 2.2 (1.4–3.4), 4.7 (4.0–5.6), 6.0 (4.1–9.0), 4.1 (3.1–5.6) and 5.4 (3.6–8.0) in lithium, valproate, olanzapine, quetiapine and risperidone exposure groups, respectively. In particular, exposure to olanzapine was associated with a significantly higher rate of natural-cause mortality than lithium, with an adjusted HR of 3.04 (1.54–6.00). Regarding unnatural-cause mortality, the incidence rate in lithium, valproate, olanzapine, quetiapine and risperidone exposure groups were 1.5 (0.9–2.6), 1.8 (1.4–2.4), 2.6 (1.4–4.8), 1.6 (1.0–2.6) and 5.0 (3.3–7.5), respectively. The risperidone exposure group demonstrated a significantly increased risk of unnatural-cause mortality risk compared to the lithium group (HR: 3.33 [1.62–6.86]). Kaplan–Meier curves showing survival rates of five exposure groups are shown in Fig. 1. Findings of the additional analyses of cumulative exposure to studied mood stabilizers ≥90 days, ≥180 days, ≥365 days and ≥730 days (Supplementary Table S4) were consistent with those of the primary analyses. For the analyses restricting to patients with comparatively short duration of exposure to studied mood stabilizers, olanzapine remained significantly associated with increased risk of natural-cause mortality in exposure <180 days and <365 days (but not <90 days) relative to lithium. Association between risperidone and elevated unnatural-cause mortality risk was not observed compared to lithium in all of the three short exposure durations.

Sensitivity analyses restricting to patients with length of cumulative exposure to the specified mood stabilizer ≥90 days and its MPR ≥90% yielded a similar pattern of mortality risk across the five exposure groups (Supplementary Table S6). In the other two sets of sensitivity analyses which included (i) patients with MPR of the specified mood stabilizer ≥80% and the other mood stabilizers <20%, and (ii) patients with mood-stabilizer monotherapy, we observed that olanzapine and risperidone were both associated with increased risk of all-cause and natural-cause mortality (relative to lithium group), and the association between risperidone and unnatural-cause mortality risk became non-significant (Supplementary Tables S7 and S8).

## Discussion

To our knowledge, this investigation is one of the few population-based cohort studies comprehensively examining risk of all-, natural- and unnatural-cause mortality associated with mood stabilizers, and the first study evaluating risk of natural-cause mortality in relation to individual SGA agents relative to lithium in BD patients. We had employed PS weighting models to optimize control for confounding factors including chronic physical comorbidities, substance and alcohol use disorders and the use of other psychotropic medications to delineate the independent mortality risk of BD patients in mood-stabilizer exposure groups. Our data showed that olanzapine and risperidone users both had a significantly higher rate of all-cause mortality than patients in lithium-treated group. We also observed that olanzapine was associated with elevated risk of natural-cause mortality, while risperidone was related to increased risk of unnatural-cause mortality. There were no significant differences between lithium-, valproate- and quetiapine-treated groups in risk of all-, natural- and unnatural-cause mortality.

Specifically, findings of the primary analyses revealed that olanzapine and risperidone were related to significantly higher risk of natural- and unnatural-cause mortality than lithium, respectively. The significant association between olanzapine and increased natural-cause mortality was consistently affirmed in additional analyses on various lengths of cumulative exposure (except short exposure of <90 days) as well as three sets of sensitivity analyses. The relationship between risperidone and a higher rate of mortality due to unnatural causes (relative to lithium) did not reach statistical significance in additional analyses on short exposure duration (<365 days, albeit remained significant for longer exposure of ≥1 and ≥2 years) and in the sensitivity analyses restricting to low MPR of other mood stabilizers and monotherapy (i.e., risperidone-only), precluding definitive conclusion in this respect. The markedly higher risk estimate for unnatural-cause mortality in the risperidone-treated group than other mood-stabilizer exposure groups in three sets of sensitivity analyses, however, constituted a signal that warranted further investigation. A Swedish study comparing mortality risk of BD patients treated with individual SGA agents to those treated with lithium also revealed that olanzapine and risperidone were both associated with modestly increased mortality risk (Crump *et al.*, 2013). The excess mortality related to olanzapine and risperidone compared to lithium may have several possible explanations. First, a large body of evidence demonstrated increased prevalence of physical diseases such as metabolic syndrome, diabetes and ischemic heart diseases in individuals treated with antipsychotics, especially SGAs (Pillinger *et al.*, 2020). In particular, among SGAs, olanzapine has been shown to exhibit the worst metabolic profiles (Citrome *et al.*, 2013; Galling *et al.*, 2016; Li *et al.*, 2019; Pillinger *et al.*, 2020). Conversely, lithium may confer beneficial effect on physiological functions such as maintaining vascular elasticity by facilitating elastin biosynthesis (Xu *et al.*, 2021). Our findings of lack of significant difference between quetiapine and lithium in terms of the association with natural-cause (and all-cause) mortality risk, however, were at odds with the results of olanzapine and risperidone. Further investigation is required to verify our findings in this respect and to delineate factors attributable to potential differential mortality risk between individual SGAs in BD. Second, owing to the narrow therapeutic index, BD patients treated with lithium receive close medical surveillance via specialist psychiatric services which offer regular clinical monitoring and blood tests on lithium serum level as well as other bodily functions for potential physical complications (e.g., thyroid and renal function tests). Comparatively, monitoring (with blood tests) for patients receiving valproate and antipsychotics is generally less frequent than those prescribed with lithium in our local practice. Then more intensive monitoring of lithium-treated patients may therefore increase the likelihood of earlier detection of physical conditions with subsequent interventions. Third, individuals prescribed with olanzapine or risperidone (or other antipsychotics) might represent a subgroup of BD patients with greater illness severity. For instance, olanzapine/risperidone could be commenced for treating psychotic symptoms in BD, or after a failure of initial treatment of conventional mood stabilizers (including lithium and valproate). In this context, treatment with olanzapine/risperidone would indicate the greater illness severity of the underlying BD, which in turn might be associated with a higher risk of excess mortality, i.e., illness severity as potential confounder. Notably, SGA has replaced lithium as the most-frequently prescribed mood stabilizer in the recent decades (Lyall *et al.*, 2019; Rhee *et al.*, 2020). The excess mortality related to olanzapine and risperidone relative to lithium observed in our data indicates the need for research to further clarify the factors contributing to such association, thereby facilitating the risk–benefit evaluation of SGA use as an alternative mood-stabilizing treatment in BD. Importantly, the result that lithium was related to a lower mortality risk than olanzapine and risperidone, and comparable mortality risk with quetiapine provides further evidence supporting the need to adhere to the clinical guidelines which consistently recommend lithium (and often quetiapine) as the first-line maintenance treatment for BD (Goodwin *et al.*, 2016; Grunze *et al.*, 2013; NICE, 2014; Yatham *et al.*, 2018).

Our finding showed that valproate-treated group did not differ from lithium-treated group in the risk of unnatural death. This concurs with some Collins and McFarland, 2008; Hayes *et al.*, 2016; Smith *et al.*, 2009) but not all studies (Antolín-Concha *et al.*, 2020; Crump *et al.*, 2013; Song *et al.*, 2017), which revealed that lithium was associated with a reduced suicide risk relative to valproate. Although studies which compared patients or treatment-periods with versus without mood-stabilizing medications generally reported a lower risk estimate for lithium than for valproate (i.e., relative to no treatment) (Chen *et al.*, 2023; Toffol *et al.*, 2015; Tsai *et al.*, 2016), the substantially overlapping 95% CIs for the risk estimates of lithium and valproate in fact suggested non-significant risk difference between these two mood stabilizers. Nonetheless, caution should be exercised when comparing our findings with those of prior research specifically focusing on suicide risk as our data had included all sorts of unnatural death causes, despite suicide likely being the major contributor. Previous investigation on suicide risk between lithium- and valproate-treated patients had included both completed and attempted suicides in the composite suicidal behaviour outcome (Song *et al.*, 2017). Likewise, our data revealed a comparable risk of natural deaths in lithium and valproate exposure groups. This is contrary to an earlier register-based study demonstrating a lower risk of natural-cause mortality in BD patients treated with lithium than those with valproate within 90 days of commencement of mood-stabilizer treatment (Smith *et al.*, 2015). However, this association became non-significant when the observation period was extended to 180 days and 365 days after treatment initiation (Smith *et al.*, 2015). In fact, in our crude analyses (i.e., the unweighted sample), valproate was related to a greater likelihood of all-cause and natural-cause mortality, relative to lithium. The non-significant associations in our PS-weighted models may imply that the overall confounding effect might initially bias against valproate. The finding of a significant difference in natural-cause mortality risk between lithium and valproate in the previous study might be subject to residual confounding (Smith *et al.*, 2015). On the other hand, we had included lithium users in the lithium-treated group regardless of their subsequent discontinuation of lithium treatment, which, however, was found to be linked to increased mortality risk (Bocchetta, 2005; Müller-Oerlinghausen *et al.*, 1996; Smith *et al.*, 2015). The mortality risk of lithium exposure group may thus have been overestimated, masking the actual between-drug difference with valproate. Yet, our sensitivity analysis restricting to patients with a high degree of adherence to mood stabilizers (i.e., MPR ≥90%) yielded a similar result, indicating that such potential bias (due to raised mortality risk related to lithium discontinuation in lithium exposure group) is minimized. Of note, despite its comparable mortality risk as lithium, substantial evidence has shown that foetal exposure to valproate is associated with increased risk of major congenital malformations (Jentink *et al.*, 2010; Veroniki *et al.*, 2017) and adverse neurodevelopmental outcomes including cognitive impairment, growth retardation and behavioural disturbances such as autistic spectrum disorder and attention-deficit/hyperactivity disorder (Baldwin and Amaro, 2020; Veroniki *et al.*, 2017). In fact, various treatment guidelines have recommended valproate should be avoided in women of childbearing age (Anmella *et al.*, 2019; NICE, 2014; Yatham *et al.*, 2018). In the current study cohort, valproate-treated patients represented the largest exposure group among five studied mood stabilizers, with 58% of them being women. The comparatively high prescription rate of valproate in women with BD highlights an urgent need for a comprehensive review of local current prescribing practice and implementation of regulatory measures to enhance adherence to clinical guidelines to minimize valproate use in female BD patients during their reproductive years.

Several limitations of the study should be noted when interpreting the study results. First, data on socio-economic status, educational level, lifestyle variables such as physical activity, dietary patterns and smoking, history of abuse and previous self-harm behaviours were not adequately recorded in the medical-record database and thus were not included in the analyses for confounder adjustment. Second, similar to most other pharmacoepidemiological studies, patients’ adherence to prescribed mood stabilizers was derived from dispensing records, which may overestimate the actual intake of medications in the study cohort. Third, medication dose of mood stabilizers was not available, precluding the evaluation on the dose-dependent effect of mood stabilizers and its potential association with incremental mortality risk. Fourth, missing data on patients’ death causes may compromise the accuracy in evaluating natural- and unnatural-cause mortality. Fifth, as information on specific natural and unnatural causes was not available, we were not able to investigate the mortality risk in relation to more specific death causes such as cardiovascular-mortality and suicide. Sixth, the study data did not contain more specific illness-related information denoting disorder subtypes (type I or II), clinical polarity, presence of psychotic features and symptom severity of BD (though we used history of psychiatric admission as a proxy indicator of illness severity), failure of initial pharmacological treatment or treatment refractoriness or other psychiatric comorbidities (e.g., anxiety disorders). Hence, we were not able to explore whether the observed associations of mortality risk with mood-stabilizer exposure are similar in relation to the specific illness-related features of BD. Seventh, information about exposure to benzodiazepines, which has been shown to be associated with elevated mortality risk in patients with schizophrenia in previous research (Fontanella *et al.*, 2016; Tiihonen *et al.*, 2016) (albeit with some evidence suggesting no or minimal increased mortality risk associated with benzodiazepine use in adults [Patorno *et al.*, 2017]), was unavailable in our dataset, precluding us from adjusting for its potential confounding effect in our BD cohort. Of note, our PS weighting model, incorporating a wide array of potential confounding variables including psychotropics other than the five studied mood stabilizers (e.g., antidepressants, other mood-stabilizing anticonvulsants and other antipsychotics), yielded satisfactory post-weighting between-group balance. Notwithstanding, future research should take into consideration potential confounding effect of benzodiazepine use in mortality risk analysis for BD patients, especially those with chronic, high-dose utilization patterns. Eighth, the study dataset did not include information about formulations of antipsychotics, thus analyses examining the potential differential associations of oral vs. long-acting injectable antipsychotics with mortality risk could not be performed. Ninth, patients’ data were retrieved from medical-record database of public healthcare services managed by HA, and patients who were under private psychiatric care were not included in the study. However, HA is the predominant provider of psychiatric services to individuals with severe mental disorders in HK, and hence the risk of selection bias or missing treated cases of BD was minimized.

In conclusion, this population-based cohort study showed that BD patients in olanzapine and risperidone exposure groups had significantly higher mortality risk than those in lithium exposure group, while lithium, valproate and quetiapine were associated with comparable mortality risk. The association between olanzapine and an increased risk of natural-cause mortality relative to lithium was consistently affirmed in sensitivity analyses. Our findings thus provide evidence further supporting the adherence to treatment guidelines recommending lithium as the first-line maintenance treatment for BD. More research examining underlying factors or mechanisms that contribute to excess mortality related to individual SGA agents relative to lithium, with adequate sample size and data on more specific natural and unnatural death causes, is required to provide clinically useful data to inform risk-benefit evaluation on the use of these alternative mood stabilizers to improve treatment outcome and to minimize avoidable premature mortality in BD patients.

## Supporting information

Chan et al. supplementary material Chan et al. supplementary material

## Data Availability

The data that support the findings of this study are available from the corresponding author upon reasonable request.

## Supplementary material

The supplementary material for this article can be found at https://doi.org/10.1017/S2045796024000337.
