# Supplementary material for: Mortality risk and mood stabilizers in bipolar disorder: a propensity-score-weighted population-based cohort study in 2002–2018
Source: Epidemiol Psychiatr Sci. 2024 May 23;33:e31. doi: 10.1017/S2045796024000337 (PMC11362685; doi:10.1017/S2045796024000337)
Supplement: Chan et al. supplementary material [file S2045796024000337sup001.docx]

**Supplementary Materials**

1. **Table S1.** Prevalence of mood stabilizers in the whole BD population, incident BD population and the current study sample
2. **Fig. S1** Absolute standardized mean differences among mood-stabilizer exposure groups before and after propensity-score weighting
3. **Fig. S2** Absolute standardized mean differences among mood-stabilizer exposure groups before and after propensity-score weighting in sensitivity analysis with cumulative exposure ≥ 90 days and medication possession ratio ≥ 90%
4. **Fig. S3** Absolute standardized mean differences among mood-stabilizer exposure groups before and after propensity-score weighting in sensitivity analysis with medication possession ratios of specified mood stabilizer ≥ 80% and other mood stabilizers < 20%
5. **Fig. S4.** Absolute standardized mean differences among mood-stabilizer exposure groups before and after propensity-score weighting in sensitivity analysis with monotherapy
6. **Table S2.** ICD9-CM and ICD10 codes for diagnoses of physical diseases and psychiatric disorders
7. **Table S3.** Cumulative exposure duration of mood stabilizers in each mood-stabilizer exposure group.
8. **Table S4.** Mortality risk of mood-stabilizer exposure groups associated with cumulative exposure duration of ≥ 90 days, ≥ 180 days, ≥ 365 days or ≥ 730 days
9. **Table S5.** Mortality risk of mood-stabilizer exposure groups associated with short exposure duration
10. **Table S6.** Mortality risk of mood-stabilizer exposure groups in sensitivity analyses with cumulative exposure ≥ 90 days and medication possession ratio ≥ 90%
11. **Table S7.** Mortality risk of mood-stabilizer exposure groups in sensitivity analyses with medication possession ratios of specified mood stabilizer ≥ 80% and other mood stabilizers < 20%
12. **Table S8.** Mortality risk of mood-stabilizer exposure groups in sensitivity analyses with monotherapy

| **Table S1.** Prevalence of mood stabilizers prescribed in the whole BD cohort, incident BD cohort and the current study cohort | | | |
| --- | --- | --- | --- |
|  | Whole BD cohort (*n*=12795) | Incident BD cohort (*n*=9094) | Current study cohort (*n*=8137) |
| Mood stabilizers | Prevalence (%) | | |
| Conventional mood stabilizers | | |  |
| Lithium | 4464 (34.9) | 2459 (27.0) | 2459 (30.2) |
| Valproate | 8510 (66.5) | 5844 (64.3) | 5844 (71.8) |
| Lamotrigine | 1797 (14.0) | 1300 (14.3) | 1220 (15.0) |
| Carbamazepine | 1031 (8.1) | 365 (4.0) | 344 (4.2) |
| Second-generation antipsychotics | | |  |
| Quetiapine | 6603 (51.6) | 4854 (53.4) | 4854 (59.7) |
| Risperidone | 4831 (37.8) | 3384 (37.2) | 3384 (41.6) |
| Olanzapine | 4134 (32.3) | 2727 (30.0) | 2727 (33.5) |
| Aripiprazole | 2039 (15.9) | 1542 (17.0) | 1479 (18.2) |
| Paliperidone | 791 (6.2) | 463 (5.1) | 459 (5.6) |
| Clozapine | 392 (3.1) | 135 (1.5) | 135 (1.7) |
| First-generation antipsychotics | | |  |
| Haloperidol | 4057 (31.7) | 2186 (24.0) | 1977 (24.3) |
| Sulpiride | 2045 (16.0) | 1141 (12.5) | 1058 (13.0) |
| Trifluoperazine | 2085 (16.3) | 1067 (11.7) | 990 (12.2) |
| Chlorpromazine | 2501 (19.5) | 896 (9.9) | 836 (10.3) |
| Perphenazine | 716 (5.6) | 414 (4.6) | 403 (5.0) |

Note: BD: Bipolar disorder.


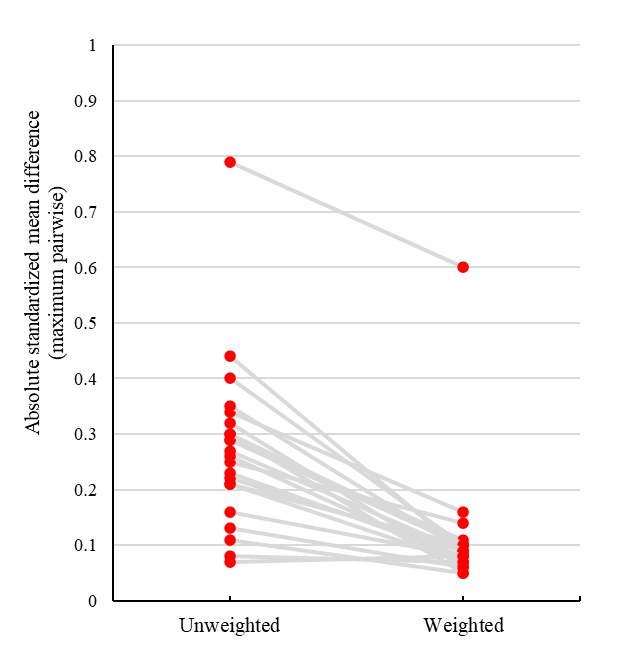


**Fig. S1** Absolute standardized mean differences among mood-stabilizer exposure groups before and after propensity-score weighting (with < 0.2 indicating good balance of covariates).

**Fig. S2** Absolute standardized mean differences among mood-stabilizer exposure groups before and after propensity-score weighting in sensitivity analysis with cumulative exposure ≥ 90 days and medication possession ratio ≥ 90% (with < 0.2 indicating good balance of covariates).


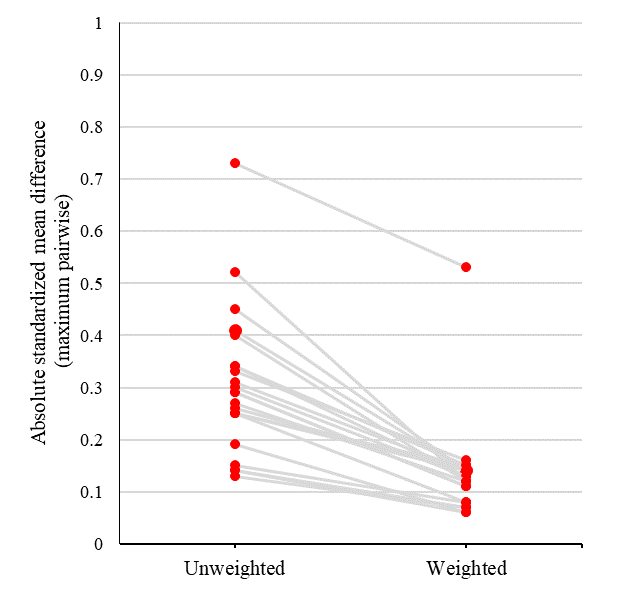

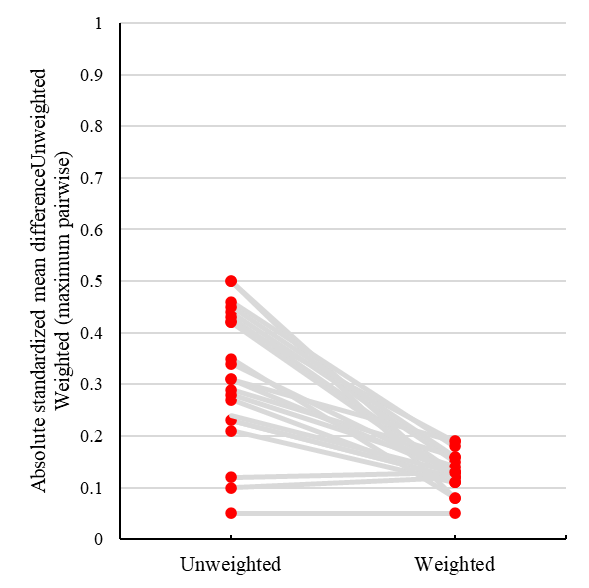


**Fig. S3** Absolute standardized mean differences among mood-stabilizer exposure groups before and after propensity-score weighting in sensitivity analysis with medication possession ratios of specified mood stabilizer ≥80% and other mood stabilizers <20% (with <0.2 indicating good balance of covariates).

**Fig. S4** Absolute standardized mean differences among mood-stabilizer exposure groups before and after propensity-score weighting in sensitivity analysis with monotherapy (with <0.2 indicating good balance of covariates).


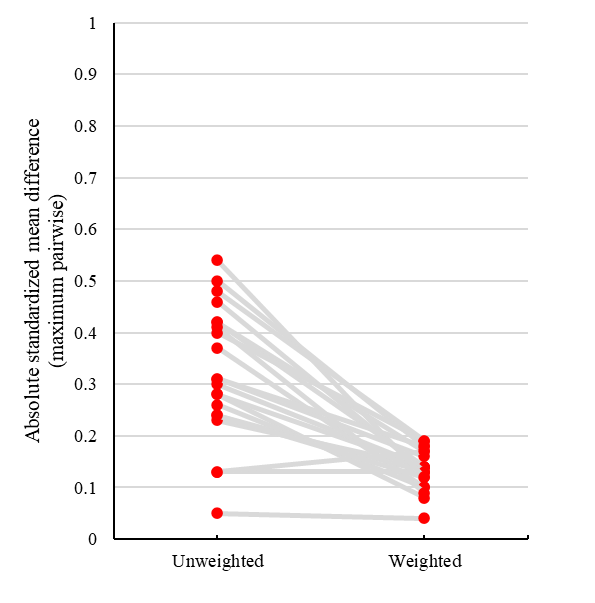


**Table S2**. ICD9-CM and ICD10 codes for diagnoses of physical diseases and psychiatric disorders

| Items | Operational definition |
| --- | --- |
| Physical diseases | ICD9-CM codes (unless otherwise specified) |
| Cerebrovascular diseases | 430–438 |
| Hemiplegia or paraplegia | 344.1, 342–342.9 |
| Myocardial infarction | 410–410.9, 412 |
| Congestive heart failure | 428–428.9 |
| Peripheral vascular diseases | 443.9, 441–441.9, 785.4, V34.4, Procedure code: 38.48 |
| Respiratory diseases | 490–496, 500–505, 506.4 |
| Peptic ulcer diseases | 531–534.9, 531.4–531.7, 532.4–542.7, 533.4–533.7, 534.4–534.7 |
| Mild liver diseases | 571.2, 571.5, 571.6, 571.4–571.49 |
| Moderate or severe liver diseases | 572.2–572.8 |
| Renal diseases | 582–582.9, 583–583.7, 585, 586, 588–588.9 |
| Rheumatological disease | 710.0, 710.1, 710.4, 714.0–714.2, 714.81, 725 |
| Any malignancy | 140–172.9, 174–195.8, 200–208.9 |
| Metastatic solid tumor | 196–199.1 |
| HIV or AIDS | 042–044 |
| Hypertension | 401, 405 |
| Diabetes mellitus | 250 |
| Dyslipidemia | 272 |
| Epilepsy | 345 |
| Psychiatric disorders | ICD10 codes |
| Bipolar disorder | F30, F31 |
| Schizophrenia or schizoaffective disorder | F20, F25 |
| Alcohol use disorder | F10 |
| Substance use disorder | F11–19 |

| **Table S3.** Cumulative exposure duration of mood stabilizers in each mood-stabilizer exposure group | | | | | |
| --- | --- | --- | --- | --- | --- |
| Mood stabilizers, days (mean, SD) | Lithium ^a^  (*n* = 1028) | Valproate ^b^  (*n* = 3580) | Olanzapine ^c^  (*n* = 797) | Quetiapine ^d^  (*n* = 1975) | Risperidone ^e^  (*n* = 757) |
| Traditional mood stabilizer | | |  |  |  |
| Valproate | 402 (904) | 2160 (1633) | 389 (763) | 405 (800) | 480 (934) |
| Lithium | 2385 (1764) | 195 (663) | 231 (685) | 160 (553) | 119 (499) |
| Lamotrigine | 198 (695) | 120 (517) | 163 (536) | 240 (688) | 92 (468) |
| Carbamazepine | 111 (592) | 32 (297) | 24 (214) | 54 (383) | 50 (375) |
| Second-generation antipsychotics | | |  |  |  |
| Quetiapine | 412 (870) | 439 (878) | 121 (325) | 1306 (1180) | 83 (332) |
| Risperidone | 181 (555) | 232 (623) | 67 (212) | 55 (227) | 1154 (1280) |
| Olanzapine | 351 (823) | 249 (645) | 1192 (1184) | 51 (208) | 24 (109) |
| Aripiprazole | 106 (372) | 119 (414) | 114 (358) | 71 (285) | 82 (363) |
| Paliperidone | 21 (140) | 30 (183) | 15 (96) | 15 (123) | 23 (157) |
| Clozapine | 44 (346) | 25 (242) | 9 (110) | 3 (70) | 12 (156) |
| First-generation antipsychotics | | |  |  |  |
| Haloperidol | 182 (711) | 156 (609) | 35 (203) | 42 (289) | 46 (283) |
| Sulpiride | 108 (527) | 113 (520) | 48 (334) | 41 (273) | 25 (178) |
| Trifluoperazine | 103 (545) | 111 (566) | 36 (272) | 24 (204) | 33 (217) |
| Chlorpromazine | 160 (738) | 90 (539) | 13 (125) | 25 (221) | 43 (346) |
| Perphenazine | 30 (245) | 29 (204) | 10 (104) | 13 (156) | 18 (157) |
| Note: SD, standard deviation.  ^a^ In lithium-exposure group, median cumulative duration was 3447 days for lithium, 5 days for valproate, and 0 day for all the other listed medications.  ^b^ In valproate-exposure group, median cumulative duration was 2632 days for valproate, 7 days for quetiapine, and 0 day for all the other listed medications.  ^c^ In olanzapine-exposure group, median cumulative duration was 1066 days for olanzapine, 29 days for valproate, and 0 day for all the other listed medications.  ^d^ In quetiapine-exposure group, median cumulative duration was1106 days for quetiapine, 7 days for valproate, and 0 day for all the other listed medications.  ^e^ In risperidone-exposure group, median cumulative duration was 881 days for risperidone, 7 days for valproate and 0 day for all the other listed medications. | | | | | |

| **Table S4.** Mortality risk of mood-stabilizer exposure groups associated with cumulative exposure duration of ≥ 90 days, ≥ 180 days, ≥ 365 days or ≥ 730 days | | | | | |
| --- | --- | --- | --- | --- | --- |
| Mortality risk | Exposure groups | | | | |
|  | Lithium | Valproate | Olanzapine | Quetiapine | Risperidone |
| Cumulative exposure duration ≥90 days | *n* = 933 | *n* = 3333 | *n* = 694 | *n* = 1732 | *n* = 577 |
| All-cause death |  |  |  |  |  |
| Number of events | 50 | 227 | 36 | 70 | 50 |
| Unadjusted HR (95% CI) | Reference | 1.44 (1.06 – 1.95) | 1.86 (1.21 – 2.86) | 1.33 (0.93 – 1.92) | 2.51 (1.69 – 3.72) |
| Adjusted HR ^a^ (95% CI) | Reference | 1.12 (0.79 – 1.58) | 2.02 (1.24 – 3.27) | 0.96 (0.64 – 1.45) | 1.91 (1.22 – 2.99) |
| Natural death |  |  |  |  |  |
| Number of events | 18 | 129 | 20 | 39 | 22 |
| Unadjusted HR (95% CI) | Reference | 2.31 (1.41 – 3.78) | 3.11 (1.64 – 5.89) | 2.21 (1.26 – 3.87) | 3.24 (1.74 – 6.06) |
| Adjusted HR ^a^ (95% CI) | Reference | 1.54 (0.86 – 2.77) | 3.19 (1.52 – 6.71) | 1.41 (0.73 – 2.72) | 1.86 (0.89 – 3.85) |
| Unnatural death |  |  |  |  |  |
| Number of events | 13 | 42 | 8 | 17 | 20 |
| Unadjusted HR (95% CI) | Reference | 1.01 (0.54 – 1.87) | 1.39 (0.57 – 3.36) | 1.09 (0.53 – 2.25) | 3.50 (1.74 – 7.06) |
| Adjusted HR ^a^ (95% CI) | Reference | 0.91 (0.47 – 1.76) | 1.39 (0.54 – 3.57) | 0.92 (0.42 – 1.99) | 3.40 (1.61 – 7.20) |
|  |  |  |  |  |  |
| Cumulative exposure duration ≥180 days | *n* = 919 | *n* = 3207 | *n* = 636 | *n* = 1631 | *n* = 512 |
| All-cause death |  |  |  |  |  |
| Number of events | 47 | 213 | 33 | 64 | 41 |
| Unadjusted HR (95% CI) | Reference | 1.44 (1.05 – 1.97) | 1.91 (1.22 – 2.99) | 1.33 (0.91 – 1.94) | 2.37 (1.56 – 3.61) |
| Adjusted HR ^a^ (95% CI) | Reference | 1.11 (0.78 – 1.58) | 2.02 (1.22 – 3.36) | 0.95 (0.62 – 1.46) | 1.79 (1.11 – 2.90) |
| Natural death |  |  |  |  |  |
| Number of events | 17 | 124 | 18 | 37 | 19 |
| Unadjusted HR (95% CI) | Reference | 2.34 (1.41 – 3.89) | 3.06 (1.57 – 5.95) | 2.22 (1.25 – 3.96) | 3.17 (1.65 – 6.12) |
| Adjusted HR ^a^ (95% CI) | Reference | 1.52 (0.84 – 2.77) | 2.99 (1.38 – 6.51) | 1.40 (0.71 – 2.74) | 1.83 (0.85 – 3.93) |
| Unnatural death |  |  |  |  |  |
| Number of events | 13 | 36 | 7 | 14 | 15 |
| Unadjusted HR (95% CI) | Reference | 0.87 (0.46 – 1.64) | 1.31 (0.52 – 3.29) | 0.94 (0.44 – 2.00) | 2.89 (1.37 – 6.09) |
| Adjusted HR ^a^ (95% CI) | Reference | 0.78 (0.40 – 1.53) | 1.30 (0.48 – 3.49) | 0.80 (0.35 – 1.81) | 2.83 (1.26 – 6.35) |
|  |  |  |  |  |  |
| Cumulative exposure duration ≥365 days | *n* = 863 | *n* = 2983 | *n* = 562 | *n* = 1456 | *n* = 442 |
| All-cause death |  |  |  |  |  |
| Number of events | 44 | 202 | 31 | 54 | 37 |
| Unadjusted HR (95% CI) | Reference | 1.47 (1.06 – 2.04) | 2.06 (1.30 – 3.27) | 1.26 (0.84 – 1.88) | 2.43 (1.57 – 3.76) |
| Adjusted HR ^a^ (95% CI) | Reference | 1.12 (0.78 – 1.63) | 2.11 (1.25 – 3.56) | 0.88 (0.56 – 1.38) | 1.81 (1.09 – 2.99) |
| Natural death |  |  |  |  |  |
| Number of events | 17 | 119 | 17 | 33 | 18 |
| Unadjusted HR (95% CI) | Reference | 2.27 (1.37 – 3.78) | 3.06 (1.56 – 6.01) | 2.06 (1.15 – 3.72) | 3.17 (1.63 – 6.15) |
| Adjusted HR ^a^ (95% CI) | Reference | 1.47 (0.80 – 2.68) | 2.89 (1.31 – 6.36) | 1.27 (0.64 – 2.53) | 1.80 (0.83 – 3.92) |
| Unnatural death |  |  |  |  |  |
| Number of events | 11 | 33 | 7 | 11 | 13 |
| Unadjusted HR (95% CI) | Reference | 0.95 (0.48 – 1.88) | 1.67 (0.64 – 4.33) | 0.92 (0.40 – 2.14) | 3.16 (1.41 – 7.08) |
| Adjusted HR ^a^ (95% CI) | Reference | 0.88 (0.43 – 1.82) | 1.66 (0.60 – 4.62) | 0.83 (0.33 – 2.05) | 3.32 (1.39 – 7.94) |
|  |  |  |  |  |  |
| Cumulative exposure duration ≥730 days | *n* = 769 | *n* = 2630 | *n* = 437 | *n* = 1169 | *n* = 358 |
| All-cause death |  |  |  |  |  |
| Number of events | 36 | 171 | 24 | 37 | 27 |
| Unadjusted HR (95% CI) | Reference | 1.58 (1.10 – 2.26) | 2.25 (1.34 – 3.78) | 1.19 (0.75 – 1.88) | 2.46 (1.49 – 4.06) |
| Adjusted HR ^a^ (95% CI) | Reference | 1.29 (0.87 – 1.93) | 2.40 (1.36 – 4.24) | 0.87 (0.52 – 1.45) | 1.98 (1.12 – 3.50) |
| Natural death |  |  |  |  |  |
| Number of events | 15 | 98 | 13 | 22 | 14 |
| Unadjusted HR (95% CI) | Reference | 2.20 (1.28 – 3.79) | 3.09 (1.47 – 6.51) | 1.76 (0.91 – 3.41) | 3.16 (1.52 – 6.56) |
| Adjusted HR ^a^ (95% CI) | Reference | 1.51 (0.80 – 2.84) | 3.11 (1.36 – 7.13) | 1.11 (0.52 – 2.35) | 1.96 (0.85 – 4.51) |
| Unnatural death |  |  |  |  |  |
| Number of events | 8 | 30 | 5 | 9 | 9 |
| Unadjusted HR (95% CI) | Reference | 1.22 (0.56 – 2.66) | 1.89 (0.62 – 5.81) | 1.16 (0.44 – 3.01) | 3.38 (1.30 – 8.77) |
| Adjusted HR ^a^ (95% CI) | Reference | 1.23 (0.54 – 2.80) | 2.06 (0.63 – 6.78) | 1.12 (0.40 – 3.08) | 3.72 (1.32 – 10.48) |
| Note: CI, confidence intervals; HR, hazard ratio; SD, standard deviation.  ^a^ The model was propensity score-weighted. | | | | | |

| **Table S5.** Mortality risk of mood-stabilizer exposure groups with short exposure duration | | | | | |
| --- | --- | --- | --- | --- | --- |
| Mortality risk | Exposure groups | | | | |
|  | Lithium | Valproate | Olanzapine | Quetiapine | Risperidone |
| Cumulative exposure duration <90 days | *n* = 63 | *n* = 247 | *n* = 103 | *n* = 243 | *n* = 180 |
| All-cause death |  |  |  |  |  |
| Number of events | 5 | 24 | 9 | 10 | 7 |
| Unadjusted HR (95% CI) | Reference | 1.47 (0.56 – 3.86) | 1.97 (0.65 – 5.97) | 0.93 (0.31 – 2.75) | 0.73 (0.23 – 2.31) |
| Adjusted HR ^a^ (95% CI) | Reference | 1.48 (0.54 – 4.07) | 2.67 (0.81 – 8.87) | 0.94 (0.29 – 3.08) | 0.85 (0.25 – 2.88) |
| Natural death |  |  |  |  |  |
| Number of events | 2 | 10 | 4 | 5 | 3 |
| Unadjusted HR (95% CI) | Reference | 1.72 (0.38 – 7.86) | 3.30 (0.59 – 18.37) | 1.77 (0.34 – 9.33) | 1.02 (0.17 – 6.20) |
| Adjusted HR ^a^ (95% CI) | Reference | 1.74 (0.39 – 7.89) | 5.13 (0.90 – 29.20) | 1.93 (0.35 – 10.64) | 1.21 (0.19 – 7.86) |
| Unnatural death |  |  |  |  |  |
| Number of events | 1 | 11 | 2 | 0 | 3 |
| Unadjusted HR (95% CI) | Reference | 2.87 (0.37 – 22.26) | 1.43 (0.13 – 15.82) | NA | 1.07 (0.11 – 10.28) |
| Adjusted HR ^a^ (95% CI) | Reference | 2.09 (0.26 – 16.51) | 1.17 (0.10 – 14.16) | NA | 0.92 (0.10 – 9.00) |
|  |  |  |  |  |  |
| Cumulative exposure duration <180 days | *n* = 109 | *n* = 373 | *n* = 161 | *n* = 344 | *n* = 245 |
| All-cause death |  |  |  |  |  |
| Number of events | 8 | 38 | 12 | 16 | 16 |
| Unadjusted HR (95% CI) | Reference | 1.47 (0.69 – 3.16) | 1.59 (0.65 – 3.93) | 1.01 (0.43 – 2.38) | 1.15 (0.49 – 2.71) |
| Adjusted HR ^a^ (95% CI) | Reference | 1.42 (0.64 – 3.16) | 2.23 (0.85 – 5.88) | 0.97 (0.39 – 2.42) | 1.24 (0.51 – 3.04) |
| Natural death |  |  |  |  |  |
| Number of events | 3 | 15 | 6 | 7 | 6 |
| Unadjusted HR (95% CI) | Reference | 1.71 (0.49 – 5.91) | 3.06 (0.75 – 12.39) | 1.75 (0.44 – 6.90) | 1.47 (0.36 – 5.91) |
| Adjusted HR ^a^ (95% CI) | Reference | 1.82 (0.53 – 6.26) | 5.46 (1.34 – 22.19) | 1.87 (0.46 – 7.55) | 1.54 (0.37 – 6.41) |
| Unnatural death |  |  |  |  |  |
| Number of events | 1 | 17 | 3 | 3 | 8 |
| Unadjusted HR (95% CI) | Reference | 4.81 (0.64 – 36.15) | 2.37 (0.25 – 22.86) | 1.10 (0.11 – 10.58) | 3.57 (0.45 – 28.59) |
| Adjusted HR ^a^ (95% CI) | Reference | 3.52 (0.47 – 26.66) | 2.03 (0.20 – 20.49) | 0.75 (0.08 – 7.45) | 3.15 (0.39 – 25.40) |
|  |  |  |  |  |  |
| Cumulative exposure duration <365 days | *n* = 165 | *n* = 597 | *n* = 235 | *n* = 519 | *n* = 315 |
| All-cause death |  |  |  |  |  |
| Number of events | 11 | 49 | 14 | 26 | 20 |
| Unadjusted HR (95% CI) | Reference | 1.28 (0.67 – 2.47) | 1.32 (0.59 – 2.92) | 1.13 (0.55 – 2.30) | 1.18 (0.56 – 2.47) |
| Adjusted HR ^a^ (95% CI) | Reference | 1.24 (0.62 – 2.46) | 1.94 (0.82 – 4.58) | 1.14 (0.53 – 2.43) | 1.28 (0.59 – 2.79) |
| Natural death |  |  |  |  |  |
| Number of events | 3 | 20 | 7 | 11 | 7 |
| Unadjusted HR (95% CI) | Reference | 1.28 (0.67 – 2.47) | 1.32 (0.59 – 2.92) | 1.13 (0.55 – 2.30) | 1.18 (0.56 – 2.47) |
| Adjusted HR ^a^ (95% CI) | Reference | 2.32 (0.69 – 7.83) | 6.50 (1.63 – 25.97) | 2.84 (0.77 – 10.38) | 2.03 (0.51 – 8.14) |
| Unnatural death |  |  |  |  |  |
| Number of events | 3 | 20 | 3 | 6 | 10 |
| Unadjusted HR (95% CI) | Reference | 1.84 (0.55 – 6.20) | 0.85 (0.17 – 4.21) | 0.78 (0.19 – 3.12) | 1.82 (0.50 – 6.63) |
| Adjusted HR ^a^ (95% CI) | Reference | 1.45 (0.42 – 4.94) | 0.77 (0.15 – 3.98) | 0.57 (0.14 – 2.34) | 1.62 (0.44 – 5.97) |
| Note: CI, confidence intervals; HR, hazard ratio; SD, standard deviation.  ^a^ The model was propensity score-weighted. | | | | | |

| **Table S6.** Mortality risk of mood-stabilizer exposure groups in sensitivity analyses with cumulative exposure ≥ 90 days and medication possession ratio ≥ 90% | | | | | |
| --- | --- | --- | --- | --- | --- |
| Mortality risk | Exposure groups | | | | |
|  | Lithium  (*n* = 524) | Valproate  (*n* = 1873) | Olanzapine  (*n* = 362) | Quetiapine  (*n* = 974) | Risperidone  (*n* = 298) |
| All-cause death |  |  |  |  |  |
| Number of events | 24 | 128 | 18 | 29 | 26 |
| Unadjusted HR (95% CI) | Reference | 1.66 (1.08 – 2.55) | 2.33 (1.28 – 4.26) | 1.31 (0.76 – 2.24) | 3.22 (1.86 – 5.60) |
| Adjusted HR ^a^ (95% CI) | Reference | 1.28 (0.80 – 2.06) | 2.46 (1.26 – 4.80) | 0.85 (0.47 – 1.54) | 2.61 (1.40 – 4.85) |
|  |  |  |  |  |  |
| Natural death |  |  |  |  |  |
| Number of events | 8 | 69 | 10 | 15 | 11 |
| Unadjusted HR (95% CI) | Reference | 2.86 (1.38 – 5.94) | 4.46 (1.75 – 11.40) | 2.51 (1.06 – 5.91) | 4.75 (1.90 – 11.88) |
| Adjusted HR ^a^ (95% CI) | Reference | 1.69 (0.73 – 3.87) | 4.22 (1.51 – 11.80) | 1.16 (0.45 – 3.03) | 2.40 (0.86 – 6.71) |
|  |  |  |  |  |  |
| Unnatural death |  |  |  |  |  |
| Number of events | 6 | 23 | 4 | 7 | 13 |
| Unadjusted HR (95% CI) | Reference | 1.02 (0.44 – 2.36) | 1.30 (0.38 – 4.46) | 0.79 (0.27 – 2.26) | 4.55 (1.81 – 11.45) |
| Adjusted HR ^a^ (95% CI) | Reference | 0.92 (0.38 – 2.27) | 1.38 (0.36 – 5.39) | 0.77 (0.25 – 2.35) | 4.95 (1.84 – 13.32) |
| Note: CI, confidence intervals; HR, hazard ratio; SD, standard deviation.  ^a^ The model was propensity score-weighted with additional adjustment for concomitant/ sequential prescription of studied mood stabilizers other than the specified one. | | | | | |

| **Table S7.** Mortality risk of mood-stabilizer exposure groups in sensitivity analyses with medication possession ratios of specified mood stabilizer ≥ 80% and other mood stabilizers < 20% | | | | | |
| --- | --- | --- | --- | --- | --- |
| Mortality risk | Exposure groups | | | | |
|  | Lithium  (*n* = 193) | Valproate  (*n* = 892) | Olanzapine  (*n* = 134) | Quetiapine  (*n* = 625) | Risperidone  (*n* = 197) |
| All-cause death |  |  |  |  |  |
| Number of events | 11 | 86 | 9 | 27 | 15 |
| Unadjusted HR (95% CI) | Reference | 1.93 (1.03 – 3.62) | 2.36 (0.97 – 5.71) | 1.59 (0.78 – 3.24) | 2.26 (1.03 – 4.93) |
| Adjusted HR ^a^ (95% CI) | Reference | 1.82 (0.92 – 3.60) | 2.56 (1.01 – 6.51) | 1.48 (0.68 – 3.24) | 2.42 (1.00 – 5.84) |
|  |  |  |  |  |  |
| Natural death |  |  |  |  |  |
| Number of events | 4 | 47 | 7 | 20 | 9 |
| Unadjusted HR (95% CI) | Reference | 3.01 (1.09 – 8.36) | 5.67 (1.65 – 19.45) | 3.73 (1.26 – 11.01) | 4.11 (1.26 – 13.40) |
| Adjusted HR ^a^ (95% CI) | Reference | 2.54 (0.84 – 7.73) | 5.46 (1.48 – 20.10) | 2.82 (0.87 – 9.16) | 4.07 (1.10 – 15.07) |
|  |  |  |  |  |  |
| Unnatural death |  |  |  |  |  |
| Number of events | 2 | 20 | 1 | 2 | 4 |
| Unadjusted HR (95% CI) | Reference | 2.45 (0.57 – 10.50) | 1.23 (0.11 – 13.63) | 0.52 (0.07 – 3.70) | 2.84 (0.52 – 15.56) |
| Adjusted HR ^a^ (95% CI) | Reference | 1.88 (0.43 – 8.30) | 1.05 (0.08 – 13.40) | 0.34 (0.04 – 2.80) | 2.81 (0.49 – 16.07) |
| Note: CI, confidence intervals; HR, hazard ratio; SD, standard deviation.  ^a^ The model was propensity score-weighted. | | | | | |

| **Table S8.** Mortality risk of mood-stabilizer exposure groups in sensitivity analyses with monotherapy | | | | | |
| --- | --- | --- | --- | --- | --- |
| Mortality risk | Exposure groups | | | | |
|  | Lithium  (*n* = 163) | Valproate  (*n* = 776) | Olanzapine  (*n* = 104) | Quetiapine  (*n* = 523) | Risperidone  (*n* = 152) |
| All-cause death |  |  |  |  |  |
| Number of events | 9 | 71 | 7 | 26 | 13 |
| Unadjusted HR (95% CI) | Reference | 1.96 (0.98 – 3.92) | 2.49 (0.93 – 6.72) | 1.96 (0.91 – 4.23) | 2.68 (1.14 – 6.29) |
| Adjusted HR ^a^ (95% CI) | Reference | 1.99 (0.91 – 4.37) | 2.78 (0.95 – 8.17) | 1.86 (0.77 – 4.49) | 3.26 (1.21 – 8.77) |
|  |  |  |  |  |  |
| Natural death |  |  |  |  |  |
| Number of events | 4 | 38 | 6 | 19 | 8 |
| Unadjusted HR (95% CI) | Reference | 2.47 (0.88 – 6.92) | 5.34 (1.50 – 19.00) | 3.67 (1.23 – 10.90) | 4.10 (1.23 – 13.71) |
| Adjusted HR ^a^ (95% CI) | Reference | 2.50 (0.76 – 8.20) | 6.01 (1.47 – 24.64) | 3.23 (0.93 – 11.21) | 4.70 (1.17 – 18.82) |
|  |  |  |  |  |  |
| Unnatural death |  |  |  |  |  |
| Number of events | 2 | 15 | 1 | 2 | 3 |
| Unadjusted HR (95% CI) | Reference | 1.83 (0.42 – 8.01) | 1.42 (0.13 – 15.78) | 0.56 (0.08 – 4.06) | 2.44 (0.40 – 14.68) |
| Adjusted HR ^a^ (95% CI) | Reference | 1.38 (0.31 – 6.21) | 1.01 (0.08 – 12.64) | 0.37 (0.04 – 3.21) | 2.93 (0.46 – 18.69) |
| Note: CI, confidence intervals; HR, hazard ratio; SD, standard deviation.  ^a^ The model was inversely weighted by propensity score. | | | | | |
